# Human umbilical endothelial cells (HUVECs) have a sex: characterisation of the phenotype of male and female cells

**DOI:** 10.1186/s13293-014-0018-2

**Published:** 2014-12-14

**Authors:** Roberta Addis, Ilaria Campesi, Marco Fois, Giampiero Capobianco, Salvatore Dessole, Grazia Fenu, Andrea Montella, Maria Grazia Cattaneo, Lucia M Vicentini, Flavia Franconi

**Affiliations:** Department of Biomedical Sciences, University of Sassari, Sassari, Italy; National Laboratory of Gender Medicine of the National Institute of Biostructures and Biosystems, Osilo, Sassari Italy; Department of Surgical, Microsurgical and Medical Sciences, Gynaecologic and Obstetric Clinic, University of Sassari, Sassari, Italy; Department of Medical Biotechnology and Translational Medicine, University of Milano, Milano, Italy; Assessorato alle Politiche per la Persona, Region Basilicata, Italy

**Keywords:** Sex differences, HUVECs, Autophagy, Birth weight

## Abstract

**Background:**

Human umbilical endothelial cells (HUVECs) are widely used to study the endothelial physiology and pathology that might be involved in sex and gender differences detected at the cardiovascular level. This study evaluated whether HUVECs are sexually dimorphic in their morphological, proliferative and migratory properties and in the gene and protein expression of oestrogen and androgen receptors and nitric oxide synthase 3 (NOS3). Moreover, because autophagy is influenced by sex, its degree was analysed in male and female HUVECs (MHUVECs and FHUVECs).

**Methods:**

Umbilical cords from healthy, normal weight male and female neonates born to healthy non-obese and non-smoking women were studied. HUVEC morphology was analysed by electron microscopy, and their function was investigated by proliferation, viability, wound healing and chemotaxis assays. Gene and protein expression for oestrogen and androgen receptors and for NOS3 were evaluated by real-time PCR and Western blotting, respectively, and the expression of the primary molecules involved in autophagy regulation [protein kinase B (Akt), mammalian target of rapamycin (mTOR), beclin-1 and microtubule-associated protein 1 light chain 3 (LC3)] were detected by Western blotting.

**Results:**

Cell proliferation, migration NOS3 mRNA and protein expression were significantly higher in FHUVECs than in MHUVECs. Conversely, beclin-1 and the LC3-II/LC3-I ratio were higher in MHUVECs than in FHUVECs, indicating that male cells are more autophagic than female cells. The expression of oestrogen and androgen receptor genes and proteins, the protein expression of Akt and mTOR and cellular size and shape were not influenced by sex. Body weights of male and female neonates were not significantly different, but the weight of male babies positively correlated with the weight of the mother, suggesting that the mother’s weight may exert a different influence on male and female babies.

**Conclusions:**

The results indicate that sex differences exist in prenatal life and are parameter-specific, suggesting that HUVECs of both sexes should be used as an *in vitro* model to increase the quality and the translational value of research. The sex differences observed in HUVECs could be relevant in explaining the diseases of adulthood because endothelial dysfunction has a crucial role in the pathogenesis of cardiovascular diseases, diabetes mellitus, neurodegeneration and immune disease.

## Background

Endothelial cells (ECs) occur in the innermost lining of all lymphatic and blood vessels. Far from being an inert layer, the endothelium participates in a large array of physiological functions, such as the transfer of water, nutrients and leukocytes across the vascular wall, innate and acquired immunity, angiogenesis, control of vasomotor tone and maintenance of blood fluidity. Furthermore, it plays an important role in a variety of human pathologies, including atherosclerosis, diabetes, cardiovascular diseases, inflammatory and autoimmune disorders and neurodegeneration [1-4]. Notably, sex-gender differences are observed in ischemic cardiovascular diseases in women and men [5]. For example, women suffer a greater extent of endothelial and smooth muscle dysfunction compared with men [6]. Thus, it has been suggested that the endothelium might be involved in the establishment of the sex and gender differences [7] observed in the pathophysiology of the cardiovascular system [5].

Although sex and gender could affect the outcomes, interpretation and applicability of data, it is not consistently reported in cell studies [8,9], even when the effects of sex hormones are analysed [10,11]. Among sex hormones, a central role in the different sex-related incidence of cardiovascular pathologies has been attributed to oestrogens [12-14]. These hormones participate in the maintenance of proper endothelial function by increasing the activity of nitric oxide synthase 3 (NOS3) [15]. Oestrogens mediate NOS3 activation, interacting with oestrogen receptors through both genomic and non-genomic mechanisms, which involve its protein kinase B (Akt)-mediated phosphorylation [16], finally leading to the synthesis of nitric oxide (NO) with vasodilating and anti-aggregating properties [12].

Human umbilical vein endothelial cells (HUVECs) are a valuable *in vitro* model for the study of EC physiology and pathology [17,18]. Interestingly, when primary cultures of HUVECs obtained from female and male umbilical cords were studied independently, some sex differences appeared. In particular, thrombin more efficiently stimulated prostacyclin and prostaglandin E2 synthesis in HUVECs isolated from male umbilical cords (male human umbilical vein endothelial cells (MHUVECs)) than in cells obtained from female umbilical cords (female human umbilical vein endothelial cells (FHUVECs)) [19], and RLIP76, a Ral effector GTPase-activating protein, significantly altered the percentage of apoptosis only in female cells [20]. Further, sex differences have been described in rat and mouse ECs [21,22].

The aim of this study was to evaluate whether HUVECs are sexually dimorphic. For this purpose, we analysed the morphological, proliferative and migratory properties and the expression of oestrogen and androgen receptors (ERs and AR, respectively) and NOS3 in male and female cells. Because autophagic processes are influenced by sex in various cell types and tissues [23-26], we compared the degree of autophagy and the primary molecules involved in its regulation, i.e. Akt and the mammalian target of rapamycin (mTOR), in MHUVECs and FHUVECs. The results of this study might contribute to a better knowledge of the role of endothelium in the sex differences observed in the physiology and pathophysiology of the cardiovascular system.

## Methods

### Donors

Success in culturing human endothelial cells from umbilical cords depends not only on culture conditions but also on factors preceding the harvesting of the cells, such as birth weight and maternal smoking status [27,28]. Therefore, we selected umbilical cords from healthy human male and female newborns who were vaginally delivered at term (37–42 weeks) at the Obstetrics and Gynaecology Clinic, University of Sassari, from healthy non-obese and non-smoking mothers who were drug-free, with the exception of folic acid and iron supplementation. Only umbilical cords obtained from normal weight neonates were used to obtain HUVECs. The normal weight was established according to the Ines charts described by Bertino et al. [29] (2,430–4,050 g for females and 2,550–4,190 g for males, that represented the 10th and 90th centiles in Ines charts). Informed consent was obtained from the mothers of all subjects donating umbilical cords in accordance with the Declaration of Helsinki.

### Cell isolation and characterisation

Primary FHUVECs and MHUVECs were isolated by collagenase treatment (Sigma-Aldrich, Milano, Italy), as described previously by Crampton et al. [30], and cultured in plates pre-coated with 1% gelatine (Sigma-Aldrich, Milano, Italy) in M199 medium (Life Technologies, Monza, Italy) supplemented with 10% foetal bovine serum (FBS) (Life Technologies, Monza, Italy), 10% newborn calf serum (NBCS) (Life Technologies, Monza, Italy), 1% antibiotic/antimicotic (Sigma-Aldrich, Milano, Italy) and 2 mM of l-glutamine (Sigma-Aldrich, Milano, Italy) in a 5% CO_2_ humidified atmosphere. As previously described [31], cultured cells were characterised as EC by the exhibition of cobblestone morphology when they were contact-inhibited and by an evaluation of the expression of von Willebrand factor, a glycoprotein that is constitutively stored in intraendothelial Weibel-Palade granules [32].

After seeding (75,000 cells/chamber slide), the cells were washed with phosphate-buffered saline (PBS) (Sigma-Aldrich, Milano, Italy) and fixed in 4% paraformaldehyde (Sigma-Aldrich, Milano, Italy) for 5 min at room temperature (RT) and washed in PBS. Then, the cells were fixed in cold methanol (Fluka, Sigma-Aldrich, Milano, Italy) for 1 min at RT and washed in PBS before treatment with blocking solution (PBS +4% bovine serum albumin (BSA)) (Sigma-Aldrich, Milano, Italy) and 0.1% Triton X-100 (Sigma-Aldrich, Milano, Italy) for 10 min, followed by incubation with anti-von Willebrand factor antibody (Sigma-Aldrich, Milano, Italy) (1:400 dilution in blocking solution) for 60 min at RT and washed twice with PBS. The cells were then incubated with anti-rabbit secondary antibody (Sigma-Aldrich, Milano, Italy) (dilution 1:100 in blocking solution) conjugated with a fluorescent probe for 30 min in the dark and washed again. Finally, the nuclei were counterstained with DAPI (Fluka, Sigma-Aldrich, Milano, Italy) (1 μg/ml) for 4 min in the dark. After a final wash in PBS, the slides were mounted using a mounting medium for fluorescence (Vector Laboratories, Inc., Milano, Italy), and cell images were captured using a Motic E31 microscope, an in-line digital camera, and the Motic image plus software.

FHUVECs and MHUVECs were used from passages 3–5 to ensure their endothelial characteristics, and all experiments were conducted in duplicate or triplicate.

### Proliferation, viability, wound healing and chemotaxis assays

To assess proliferation, FHUVECs (*n* = 10) and MHUVECs (*n* = 7) were seeded (15,000 cells/cm^2^ in a 96-well plate) and cultured from 1 to 7 days. Cell proliferation was measured by the 3-(4,5-dimethylthiazol-2-yl)-5-(3-carboxymethoxyphenyl)-2-(4-sulfophenyl)-2*H*-tetrazolium (MTS) assay (CellTiter 96 AQ_ueous_ One Solution Cell Proliferation assay, Promega, Milano, Italy) according to the manufacturer’s instructions. Briefly, 20 μl of MTS solution was directly added to the wells, and the conversion of MTS to formazan was recorded in a plate reader (ELx 800UV, BioTek Instruments Inc., Winooski, USA) at 490 nm after 180 min of incubation at 37°C in a humidified 5% CO_2_ atmosphere.

Cell viability was determined by trypan blue exclusion using a 0.4% trypan blue solution (Sigma-Aldrich, Milano, Italy). Viable cells were counted using a Burker cell counting chamber.

For the wound-healing assay, FHUVECs (*n* = 9) and MHUVECs (*n* = 8) were grown to confluence in gelatine-coated 12-well plates in complete medium (M199 medium supplemented with 10% charcoal (Sigma-Aldrich, Milano, Italy)-stripped FBS, 10% charcoal-stripped NBCS, 1% antibiotic/antimicotic and 2 mM l-glutamine). The use of stripped sera allows removing the hormones that it contains, minimising their effects on the migratory capacity of the cells. When the cells were 90%–100% confluent, scratches were manually made in the centre of each well using a p10 pipette tip, and the wells were cultured for 24 h. Photographs were taken just after scratching and after 6, 9, 12 and 24 h of incubation at a × 4 magnification. The percentage of wound closure was calculated using the ImageProPlus software, measuring the wound area at each time point in comparison with the initial area measured at the time of the scratch. Each condition was repeated in duplicate.

For the chemotaxis assay, HUVECs were suspended in M199 medium (Life Technologies, Monza, Italy) containing 1% BSA (Sigma-Aldrich, Milano, Italy) and added to the upper chamber of a 48-well modified Boyden chamber at a density of 5.0 × 10^4^ cells/well as previously described [33]. Filters coated with 10 μg/ml of type IV collagen were placed over a bottom chamber containing 10% FBS as the attractant. After 6 h of incubation at 37°C, the cells that had migrated to the lower side of the filter were stained with Diff-Quick stain (VWR Scientific Products, Bridgeport, NJ, USA), and 5 unit fields per filter were counted using a Zeiss microscope by a scorer blind to the experimental conditions.

### Determination of cellular size and shape

The size and shape of cells [FHUVECs (*n* = 10) and MHUVECs (*n* = 10)] were evaluated on images captured by an inverted microscope (Zeiss Televal 31, Arese, Italy) at × 100 using the Labworks software (Labworks 4.0, UVP Ltd., Cambridge, UK). The cells were photographed in different days of culture (from day 0 to day 14) in at least seven random fields by placing the multiwell about in the same position. The perimeter and area were manually evaluated by an operator who was blinded to the experiment.

### Electron microscopy

FHUVECs (*n* = 5) and MHUVECs (*n* = 5) were collected and washed in PBS, centrifuged at 300 × *g* for 5 min; after a second wash in PBS, they were fixed in 2.5% glutaraldehyde (Fluka, Sigma-Aldrich, Milano, Italy) for 120 min. After fixation with 1% osmium tetroxide (Fluka, Sigma-Aldrich, Milano, Italy), the cells were dehydrated in a series of graded alcohol solutions, treated with propylene oxide and then embedded in Durcupan resin (Sigma-Aldrich, Milano, Italy). Ultrathin sections were double-stained with uranyl acetate (Sigma-Aldrich, Milano, Italy) and lead citrate (Sigma-Aldrich, Milano, Italy). Sections were examined under a Zeiss 901 EM.

### Determination of H_2_O_2_

After 72 h of incubation, the medium obtained from FHUVECs (*n* = 10) and MHUVECs (*n* = 10) (30,000 cells/cm^2^) was collected to measure H_2_O_2_ using a commercial kit (Colorimetric Hydrogen Peroxide kit, Stressgen, Milano, Italy) following the manufacturer’s instructions. The standard curve was built by a serial dilution in PBS of a H_2_O_2_ standard solution.

### Protein quantification

Total protein was measured in cell lysates prepared as previously described [34]. The protein concentrations were quantified using the Bradford Protein Assay Reagent (Sigma-Aldrich, Milano, Italy) following the manufacturer’s instructions. The quantification of protein was determined using a standard curve built with different concentrations of BSA.

### Western blots of actin, NOS3, Akt, mTOR, beclin-1, LC3-I, LC3-II, ERs and AR

For the Western blot analysis, 30 μg of solubilised protein was electrophoretically resolved by 4%–15% SDS-PAGE (100 V, 2 h, 24°C) and then transferred to a PVDF membrane (250 mA, 65 min, 4°C) using a mini-PROTEAN tetra cell system (Bio-Rad, Milano, Italy). The membranes were blocked in 5% (*w*/*v*) skim milk (Sigma-Aldrich, Milano, Italy) in Tris buffer (150 mM NaCl (Sigma-Aldrich, Milano, Italy) and 20 mM Tris–HCl (Sigma-Aldrich, Milano, Italy), pH 7.2) at 24°C for 1 h and then incubated overnight at 4°C with actin (Sigma-Aldrich, Milano, Italy) (1:1,000), NOS3 (Cell Signaling Technology, Milano, Italy) (1:1,000), Akt (Cell Signaling Technology, Milano, Italy) (1:1,000), mTOR (Cell Signaling Technology, Milano, Italy) (1:1,000), beclin-1 (Cell Signaling Technology, Milano, Italy) (1:1,000) or microtubule-associated protein 1 light chain 3 (LC3-I and LC3-II) (MBL, Milano, Italy) (1:500), ER-α (Santa Cruz Biotechnology, Segrate, Italy) (1:100), ER-β (Santa Cruz Biotechnology, Segrate, Italy) (1:100), GPER30 (K-19)-R: sc-48524-R (Santa Cruz Biotechnology, Segrate, Italy) (1:100) and AR (C-19): sc-815 (Santa Cruz Biotechnology, Segrate, Italy) (1:100) primary antibodies. After washing to remove excess primary antibody, the blots were incubated for 1 h with horseradish peroxidase (HRP)-conjugated secondary antibody (Cell Signaling Technology, Milano, Italy) (1:2,000). Antibody binding was detected using an enhanced chemiluminescence detection system (Cell Signaling Technology, Milano, Italy). The films were scanned, and the intensity of the immunoblot bands was detected with area-density software (Labworks 4.0, UVP Ltd., Cambridge, UK).

Pilot experiments showed that FHUVECs and MHUVECs did not differ in the expression of the housekeeping protein actin (Cell Signaling Technology, Milano, Italy). This reference protein was 7,708.99 ± 567.84 OD (*n* = 35) and 7,913.24 ± 633.26 OD (*n* = 31) in FHUVECs and MHUVECs, respectively. Therefore, it can be used to normalise the results obtained with other proteins.

### NOS3, ERs and AR gene expression

Total RNA was extracted using the RNeasy®Mini Kit (Qiagen, Hilden, Germany). To avoid DNA contamination of samples, a 15-min on-column incubation was carried out with DNase I (Qiagen, Hilden, Germany). Reverse transcription was performed using the SuperScript III reverse transcriptase (Life Technologies, Monza, Italy) for NOS3 and β-actin or the High Capacity cDNA Reverse Transcription Kit for oestrogen and androgen receptors, RPL30 and B2M (Applied Biosystems, CA, USA). Random RNA samples were always retro-transcribed with H_2_O instead of reverse transcriptase (rt-blanks) to check for genomic amplification during real-time PCR. The real-time PCR reactions were carried out in duplicate. cDNA (20 ng) was used as template in a total reaction volume of 10 μl. TaqMan probes were used [ribosomal protein L30 (RPL30), Hs00265497_m1; beta-2 microglobulin (β2M), Hs99999907_m1; oestrogen receptor 1 (ER-α), Hs00174860_m1; oestrogen receptor 2 (ER-β), Hs01100353_m1*; G protein-coupled oestrogen receptor (GPER), Hs01922715_s1*; androgen receptor (AR), Hs00171172_m1*; nitric oxide synthase 3 (NOS3), Hs00167166_m1; β-actin Hs99999903_m1] according to the manufacturer’s instructions.

The TaqMan probes were chosen after checking for synonyms in the databases and specificity compared to pseudogenes and isoforms. Where possible, probes straddling exon junctions were selected. Rt-PCR results for rt-blanks were always negative. The geometric mean between two housekeeping genes was used to normalise the real-time PCR results [35]: the gene that encodes Rpl30, a protein that is a component of the ribosomal 60S subunit [36], and the gene that encodes β2M, a component of MHC class I protein.

For the quantitative analysis of NOS3 gene expression, we used the ABI PrismH 7000 Sequence Detection System, SDS software version 1.2.3 (Applied Biosystems, CA, USA). The results were calculated with the 2^−ΔΔCt^ method using β-actin as an internal control.

### Statistical analysis

Data are displayed as the mean ± SEM if they followed a Gaussian distribution or as median and median absolute deviation (MAD) if they were not normally distributed. Statistical analysis was performed by comparing FHUVECs and MHUVECs with unpaired Student’s *t*-tests or paired Student’s *t*-tests when the data followed a normal distribution, using Sigma-Stat 3.1 software. Non-parametric variables were compared using the Mann–Whitney rank sum test and the Wilcoxon signed rank sum test. The distribution of samples was evaluated by the Kolmogorov-Smirnov and Shapiro tests. A *P* ≤ 0.05 was considered statistically significant. The strength of association between variables was analysed with the Pearson product moment correlation coefficient when the data were normally distributed or with the Spearman product moment correlation coefficient if the data displayed a non-Gaussian distribution. Differences in the growth rate of FHUVECs and MHUVECs were assessed using linear regression, in which slope variations were compared using a global test of coincidence.

For statistical analysis of real-time data, the Relative Expression Software Tool (REST©) with 2,000 iterations was used [37].

## Results

### Age and weight of mothers and neonates stratified by sex

Table 1 shows the age and weight of mothers and the weight of newborns stratified for sex. Mothers of both female and male babies had very similar ages and weights. The female babies weighed less than the males, but the difference did not reach statistical significance.

**Table 1:** **Age and body weight of newborns stratified by sex**

**Sex of donors**	**Age of mother (years)**	**Weight of mother(kg)**	**Body weight of neonate (kg)**
Male (*n* = 85)	30.93 ± 6.35	71.29 ± 7.67	3.36 ± 0.36
Female (*n* = 91)	31.96 ± 5.40	70.02 ± 8.32	3.24 ± 0.35

Values are reported as the mean ± SEM of the mothers’ age and weight and the neonates’ weight. *n* = sample size.

**Figure 1:**
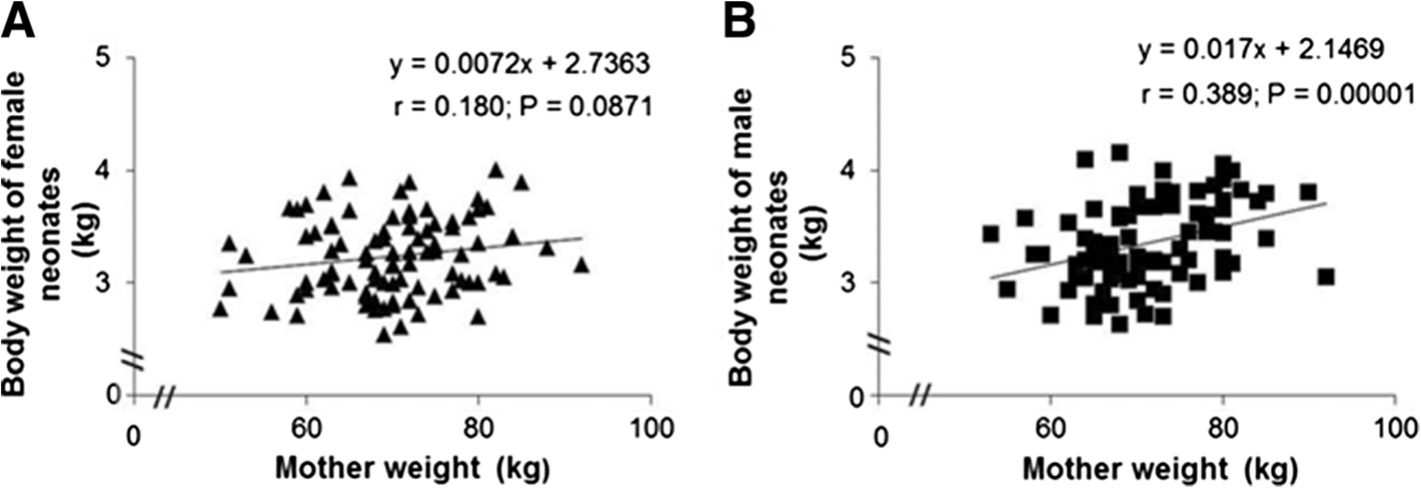
**Correlation between neonates and maternal weight (kg) stratified by sex. (A, B)** Each chart contains the equation of the line, the Spearman product moment correlation coefficient and the *P* value.

A highly significant positive correlation was found between the weight of male newborns and their mother’s weight (*r* = 0.389; *P* = 0.00001), while no significant correlation (*r* = 0.180; *P* = 0.0871) was found between the weight of female newborns and the weight of their mothers (Figure 1).

### Cell proliferation, viability, wound healing and chemotaxis assays

**Figure 2:**
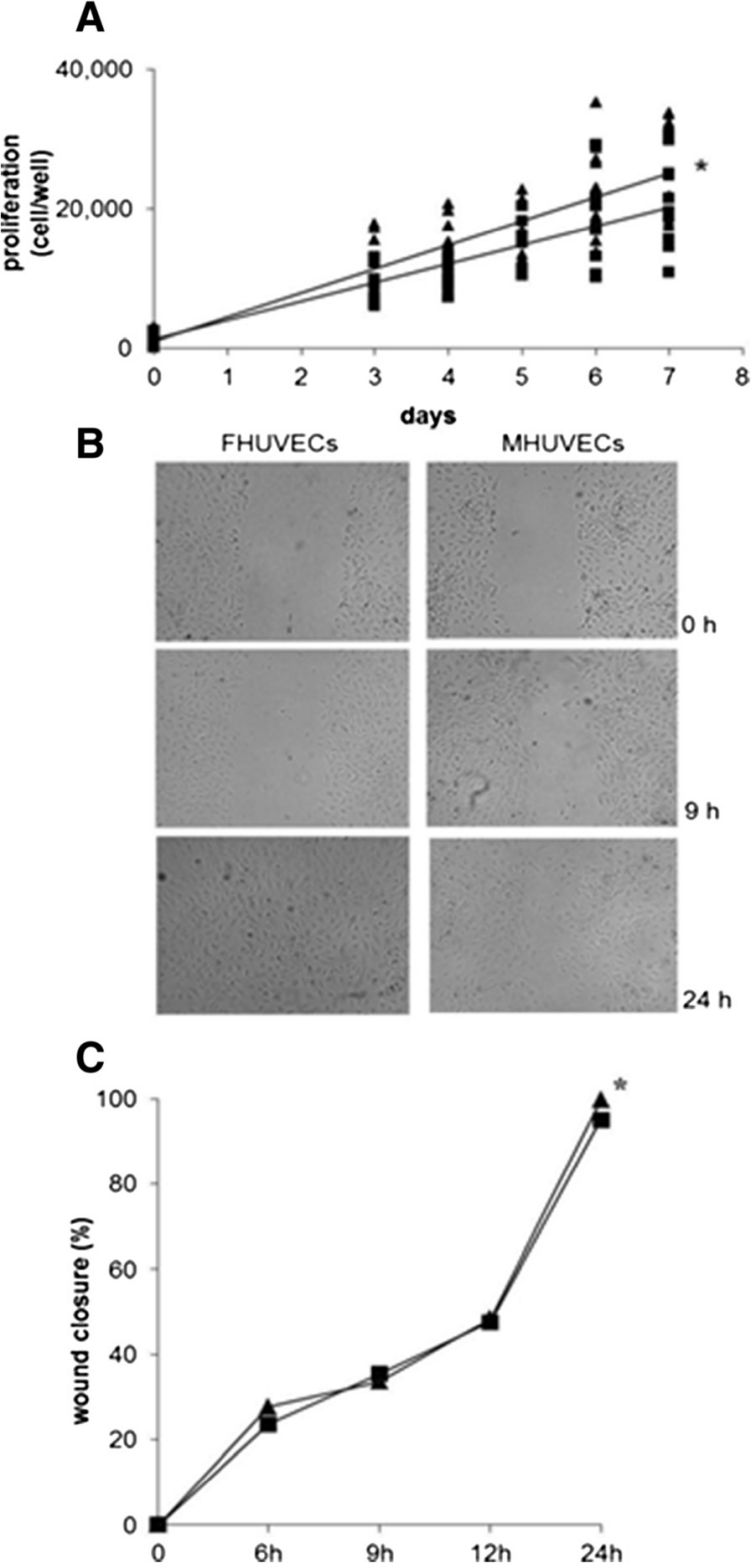
**Proliferation (A) and migration of FHUVECs and MHUVECs (B, C).** Linear regression analysis **(A)** of the proliferation of FHUVECs (▲, *n* = 10) and MHUVECs (▄, *n* = 7). *The comparison of the two slopes indicates that the slopes are different (*P* < 0.001). **(B)** Representative photographs of wound closure at 0, 9 and 24 h taken at × 4 magnification. **(C)** Mean of basal migratory capacity of MHUVECs (▄, *n* = 8) and FHUVECs (▲, *n* = 9). The data are expressed as median percentage of wound closure + MAD compared with the initial area (0 h). *P = 0.024.

Growth curves of FHUVECs (*n* = 10) and MHUVECs (*n* = 10) were conducted over a 7-day period, and the proliferation rates were calculated by plotting the number of cells against time. The plots revealed a positive linear correlation in both FHUVECs (*r* = 0.857; *P* < 0.0001) and MHUVECs (*r* = 0.837; *P* < 0.0001). The comparison of slopes (*y* = 3,452*x* + 972.8 in FHUVECs; *y* = 2,708.2*x* + 1,291.4 in MHUVECs) showed that the proliferation rate was significantly higher (*P* < 0.01) in FHUVECs than in MHUVECs (Figure 2A). The viability of FHUVECs and MHUVECs did not present any significant differences between the sexes (data not shown).

Finally, the basal wound closure capacity of FHUVECs (*n* = 9) and MHUVECs (*n* = 8), which is indicative of cell migration ability, did not diverge in the first phase of migration, but after 24 h, it was significantly higher in FHUVECs than in MHUVECs (*P* = 0.024) (Figure 2B,C). When cell motility was evaluated by chemotaxis assays in the presence of 10% FBS as an attractant, FHUVECs showed a greater tendency to migrate in comparison with MHUVECs (data not shown), although this difference did not reach statistical significance (*P* = 0.129).

### Cellular size, shape and ultrastructure differences between FHUVECs and MHUVECs

**Figure 3:**
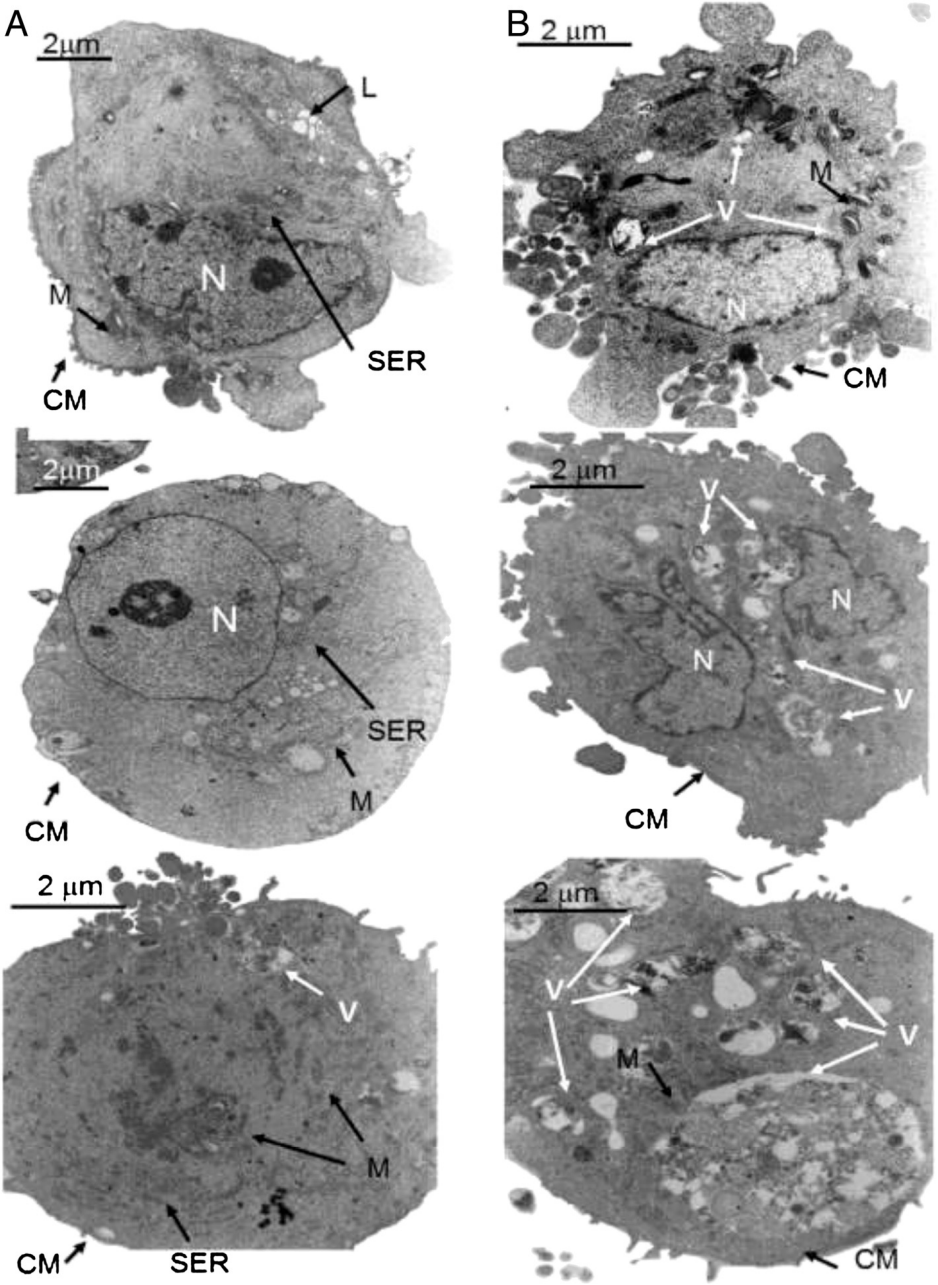
**Electron microscopic analysis of FHUVECs and MHUVECs. (** **A)** FHUVECs have little smooth endoplasmic reticulum (SER), abundant lysosomes (L) and a normal cytoplasmic membrane (CM). M and N indicate the mitochondria and nucleus, respectively. **(B)** MHUVECs have abundant autophagic vacuoles (V) in different stages of digestion and a CM with many blebs. M and N indicate the mitochondria and nucleus, respectively. **(A)** Magnification × 6,900, scale bars: 2 mm. **(B)** Magnification × 9,000, scale bars: 2 mm.

According to Martín de Llano JJ [28], blind analyses of images captured by an inverted microscope reveal that the projection area, shape and morphology of FHUVECs and MHUVECs are not significantly different (data not shown). However, transmission electron microscopy (Figure 3) revealed that FHUVECs (*n* = 5) and MHUVECs (*n* = 5) had a different ultrastructure pattern. In fact, MHUVECs showed uniformly distributed pinocytic vesicles on the cellular membrane surface, a cytoplasm with the usual organelles and one or two nuclei with nucleoli. Furthermore, the presence of swollen ribosomes that were mostly enveloped by lysosomal membranes suggested that the cells were engaged in autophagy.

Lysosomes were filled with heterogeneous material, suggesting a slow but constant activity. The cells were not in the same digestive phase: we observed both cells filled with lysosomes, which is typical of early autophagy, and cells with a dense cytoplasm and almost a complete absence of organelles, which is typical of late autophagy. The final stage of autophagy was characterised by a disappearance of cellular organelles, including the nucleus. Cellular membranes often showed breakage points, derangement of the usual double-layer structure and complete absence of vesicles. In FHUVECs, the localisation of pinocytic vesicles on the cellular surface was eccentric, and the cytoplasm contained lipid vacuoles that were absent in MHUVECs. The intermediate stages were associated with a redistribution of vesicles on the whole cellular surface and with a more electron dense cytoplasm that was primarily occupied by smooth endoplasmic reticulum. Finally, the nuclei of FHUVECs and MHUVECs had normal membranes and a single or double nucleolus with small chromatin granules.

### H_2_O_2_ production

**Figure 4:**
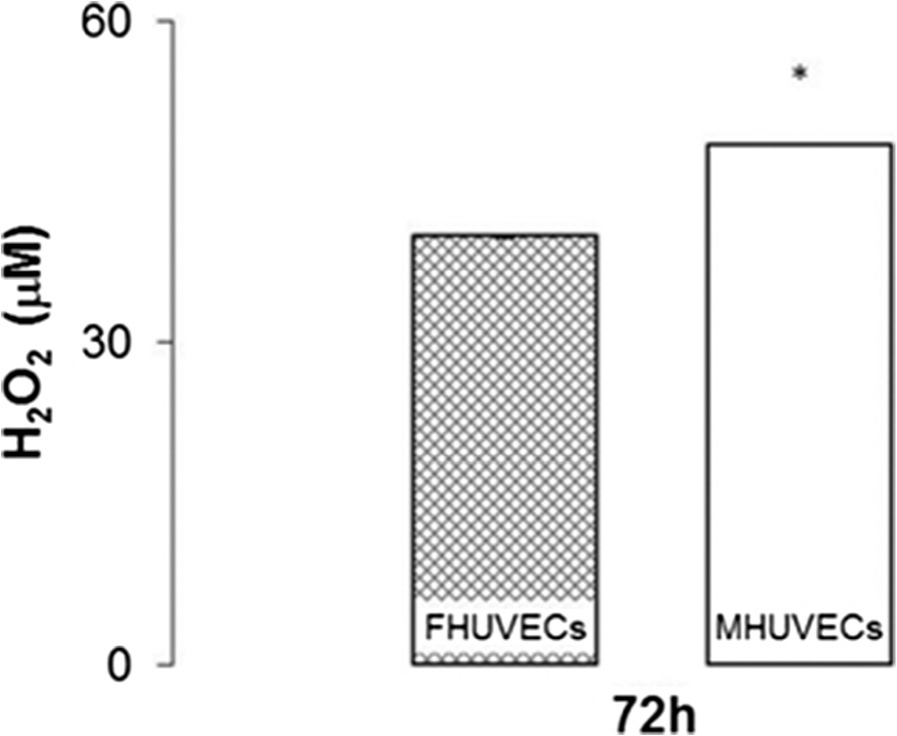
**H**_**2**_ **O**_**2**_ **levels in the supernatants obtained from FHUVECs and MHUVECs.** Values are expressed as medians + MAD of 15 independent samples at 72 h (*P = 0.026).

H_2_O_2_ is one of the most important reactive oxygen species, and its production appears to be sex-dependent in many mammalian tissues [38]. In line with previous results, Figure 4 shows that the generation of H_2_O_2_ after 72 h of incubation was significantly higher in MHUVECs (*n* = 15) than in FHUVECs (*n* = 15).

### NOS3 gene and protein expression

**Figure 5:**
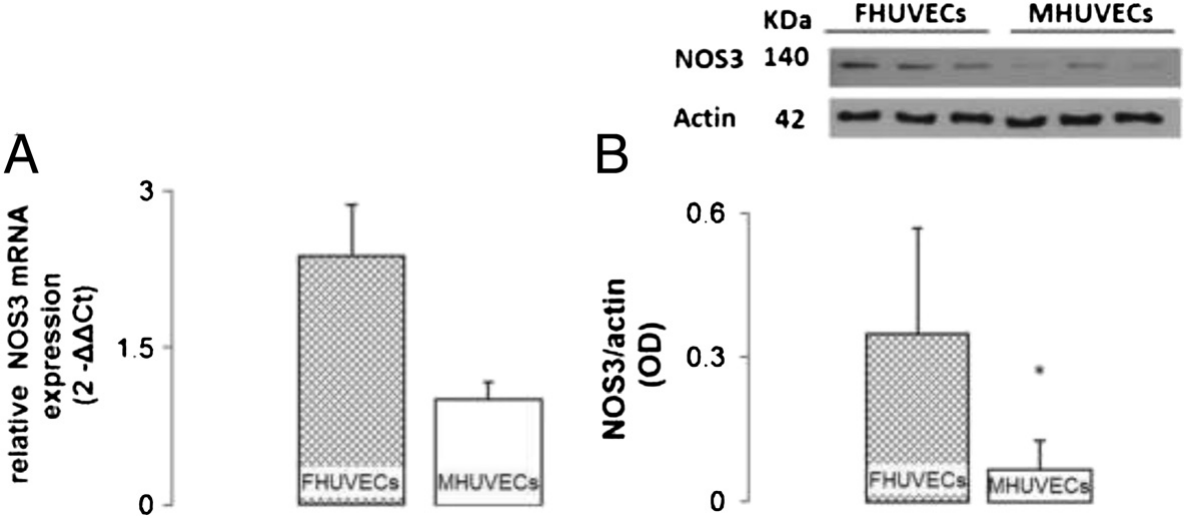
**NOS3 mRNA expression and NOS3 protein expression in FHUVECs and MHUVECs. (A)** Relative NOS3 mRNA expression. The values are expressed as the means ± SEM (*P = 0.036) of four independent experiments for each sex. **(B)** Representative Western blot and densitometric analysis of NOS3 expression. The values are expressed as medians + MAD (*P < 0.05) of 17 independent experiments for each sex normalised to actin levels.

The ability to constitutively produce NO through the activity of NOS3 represents an essential feature of ECs. We therefore evaluated NOS3 gene and protein expression by RT-qPCR and Western blot, respectively, in male and female HUVECs (RT-qPCR: FHUVECs (*n* = 4), MHUVECs (*n* = 4); protein levels: FHUVECs (*n* = 17), MHUVECs (*n* = 17)). Figure 5 shows that NOS3 was expressed at a higher level in female cells than in male cells at both the mRNA and protein levels (Figure 5A,B).

### Expression of autophagic markers: beclin-1, LC3-I and LC3-II

**Figure 6:**
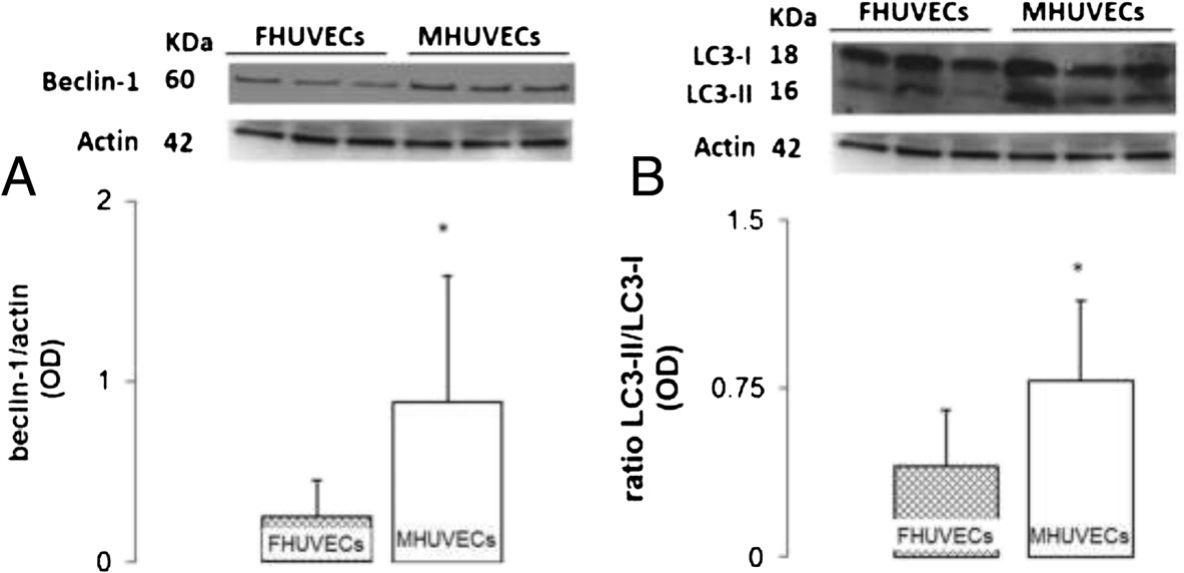
**Beclin-1 and LC3II to LC3I ratio in FHUVECs and MHUVECs. (A)** Representative Western blot and densitometric analysis of beclin-1 expression. **(B)** Ratio of LC3-II to LC3-I and representative Western blot. Values for each protein are expressed as medians + MAD (*P < 0.05) from at least 18 experiments and normalised to actin levels.

MHUVECs expressed more beclin-1 in comparison with FHUVECs (Figure 6A), while the levels of LC3-I and LC3-II were not significantly different between the groups, although in FHUVECS, the LC3-I protein showed a tendency to be higher than it was in male cells (data not shown). However, the ratio LC3-II/LC3-I, an index of autophagy [39], was significantly higher in MHUVECs than in FHUVECs (Figure 6B), thus indicating a higher constitutive autophagy in male cells, as suggested by the results of ultrastructural studies.

Finally, beclin-1 and LC3-I were positively associated only in MHUVECs (*y* = 0.9354*x* + 0.8361, *r* = 0.571, *P* = 0.0133), and LC3-I and LC3-II were not associated in both cell types (data not shown). However, a positive correlation between LC3-II and the ratio LC3-II/LC3-I was present in MHUVECs (*y* = 1.1799*x* + 0.6253, *r* = 0.688, *P* < 0.01) and in FHUVECs (*y* = 0.2122*x* + 0.3188, *r* = 0.734, *P* < 0.00001).

Autophagic processes are influenced by nutrient availability [40]. We therefore analysed whether beclin-1, LC3-I, LC3-II and the ratio LC3-II/LC3-I correlated with the body weight of neonates. Notably, only the expression of beclin-1 in MHUVECs was negatively associated with the weight of male newborns (*y* = −0.1408 + 3.3928, *r* = −0.415, *P* = 0.0285). No significant correlations were found in FHUVECs among beclin-1, LC3-I, LC3-II and the ratio LC3-II/LC3-I and the body weight of neonates.

### Expression of Akt and mTOR

**Figure 7:**
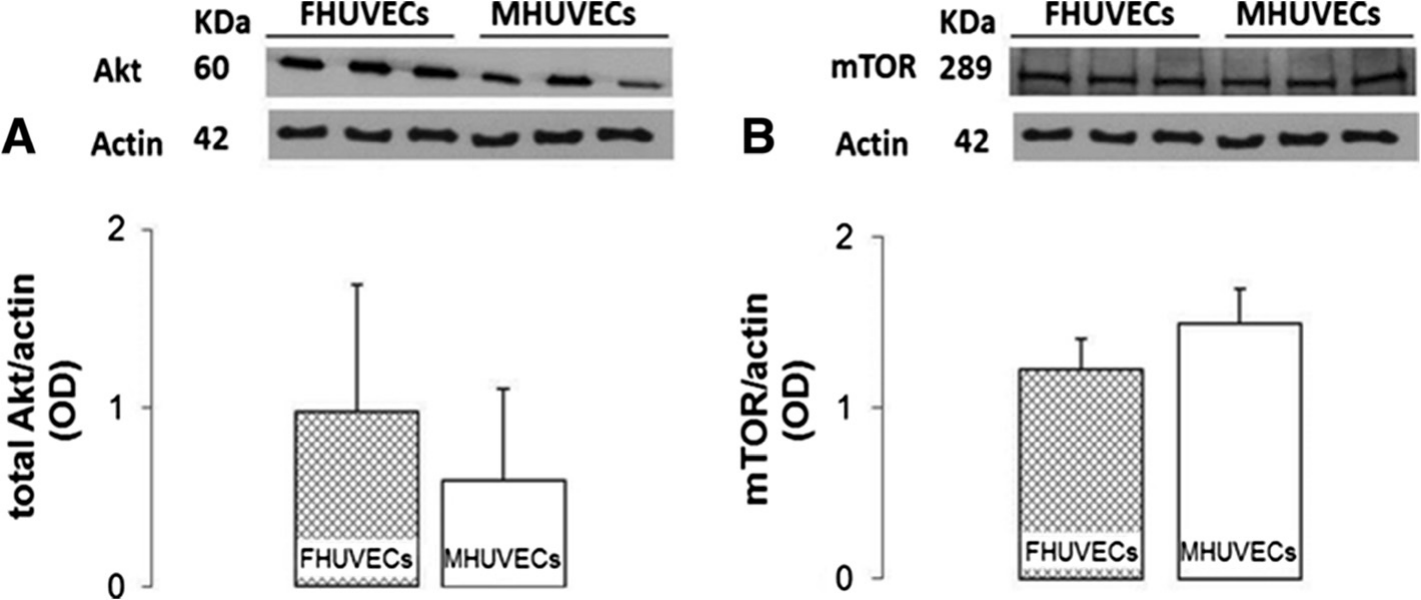
**Akt and mTOR expression levels in FHUVECs and MHUVECs. (A)** Representative Western blot and densitometric analysis of Akt expression in FHUVECs and MHUVECs. The values are expressed as medians + MAD of at least six independent experiments for each sex. Akt expression was normalised to actin levels. **(B)** Representative Western blot and densitometric analysis of mTOR expression obtained from FHUVECs and MHUVECs. The data are the means ± SEM of at least six independent experiments for each group normalised to actin.

The expression of Akt, an essential survival signalling protein, and tyrosine kinase mTOR was not different between the two cell types (Figure 7A,B).

### Expression of ERs and AR genes and protein

Whether sex differences can be due to the different expression of ERs and AR genes was explored. Both FHUVECs and MHUVECs expressed genes encoding for AR and ER. As shown in Table 2, genes for AR and classical ER (ER-α, ER-β) and GPER (formerly GPR30), which seems to mimic the protective/beneficial effects of oestrogen [41], were not different between FHUVECs and MHUVECs (Table 2).

**Table 2:** **Relative gene expression ratio between expression level in MHUVECs (** ***n*** **= 8) and FHUVECs (** ***n*** **= 9) for the oestrogen receptors (ER-α, ER-β and GPER) and the androgen receptor (AR)**

**Gene**	**Expression ratio**	**95% C.I.**	***P*** **(H1)**
ER-α	0.811	0.174–4.129	0.556
ER-β	0.900	0.415–2.299	0.564
GPER	1.058	0.145–6.225	0.880
AR	0.870	0.172–2.573	0.648

*C.I.* confidence interval, *P (H1)* probability of alternate hypothesis that the difference between males and females groups is due only to chance.

**Figure 8:**
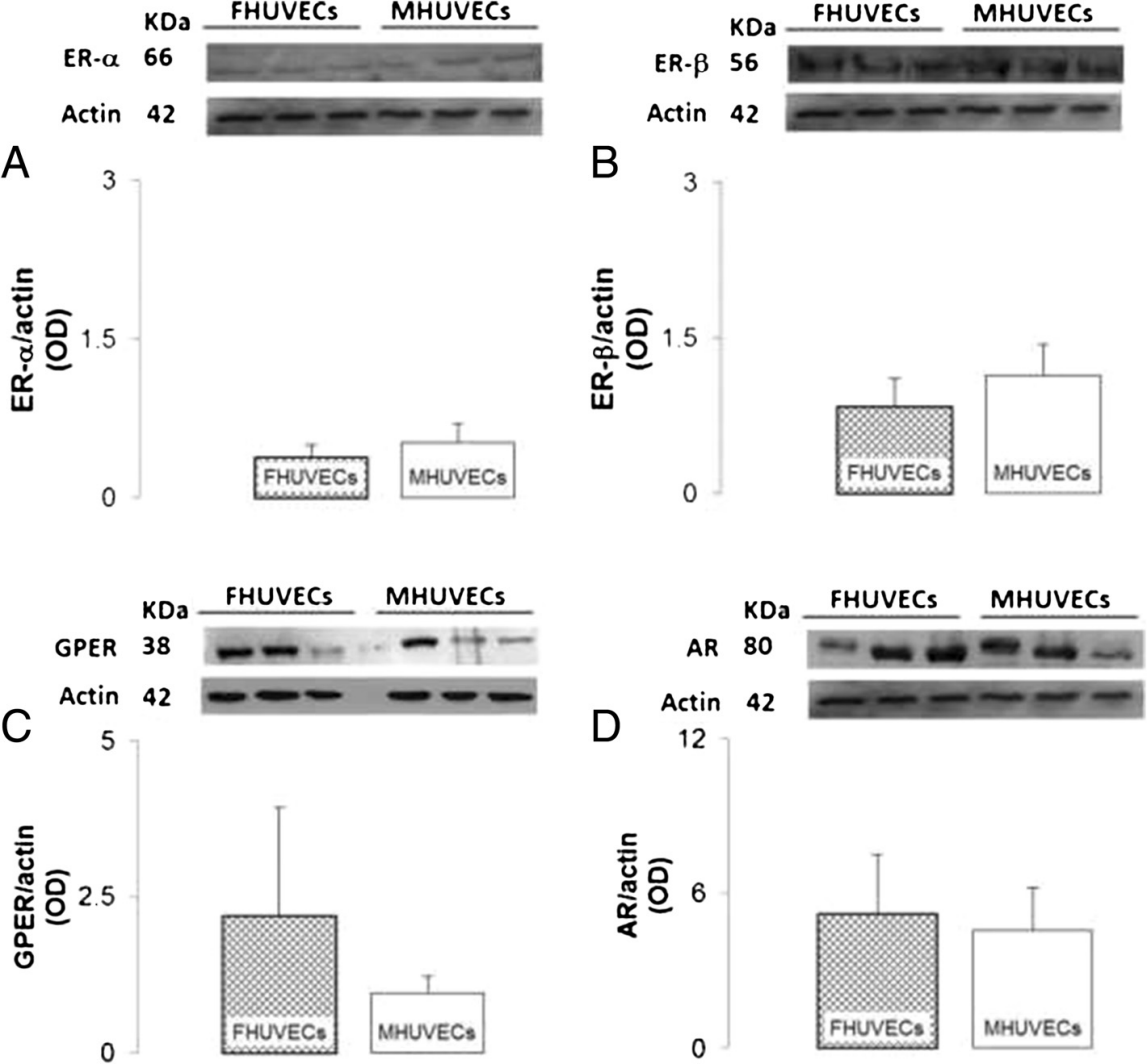
**Protein expression of ERs, GPER and AR in FHUVECs and MHUVECs. (A, B)** Representative Western blot and densitometric analysis of ER-α and ER-β expression, respectively, in FHUVECs and MHUVECs. Data are reported as the means ± SEM of at least six independent experiments for each group normalised on actin. **(C, D)** Representative Western blot and densitometric analysis of GPER and AR obtained from FHUVECs and MHUVECs. Data are reported as medians ± MAD for GPER30 and as means ± SEM for AR of at least four independent experiments for each group normalised on actin.

In the same way, the protein expression of AR, ER-α, ER-β and GPER did not present significant differences between male and female cells (Figure 8) although GPER was higher in female than in male.

## Discussion

A significant bench-to-bedside gap has been described in endothelial biomedicine [18]. Among other reasons, this could depend on the lack of studies focused on sex differences in ECs [8]. Here, we show that male and female HUVECs present some diverse characteristics that are parameter-specific. The awareness of such sex differences could help researchers understand the sexual differences observed in the incidence, presentation and outcome of cardiovascular diseases [5] and in other pathologies such as immune disorders and neurodegeneration [3,4].

Globally, our findings show that the expression of oestrogen and AR genes; the protein expression of Akt, mTOR, ER-α and ER-β, AR and GPER; and cellular size and shape are not influenced by sex in HUVECs. However, cell proliferation, migratory properties and NOS3 mRNA and protein expression are higher in FHUVECs than in MHUVECs. Conversely, beclin-1 and the LC3-II/LC3-I ratio, two widely accepted markers of autophagy, are higher in MHUVECs compared to FHUVECs, indicating that male cells are more prone to undergo autophagy than female cells. Sex differences in migratory properties, in oxidative stress and autophagy have been previously described in rat vascular smooth muscle cells [38,42].

Conflicting results for ER gene expression are obtained when experiments are performed in HUVECs without selection for cell sex. In particular, some authors have shown that HUVECs did not express the classical gene for ER-α and/or ER-β [43,44], while other authors have described the expression of both receptor mRNAs [45,46]. Here, we confirm the presence of both ER-α and ER-β, thus extending the previous knowledge and evidencing that FHUVECs and MHUVECs express not only the classical receptors ER-α and ER-β but also GPER, in a similar manner. This result is quite surprising because the ER-α and ER-β genes are differently expressed in other male and female cell types [23,42,47]. However, a similar result was reported by Kim-Schulze et al. [48] in HUVECs.

Moreover, if we considered ER protein expression, conflicting results are still reported not only when experiments are performed in HUVECs without selection of sex [45,49,50] but also when gender-related protein expression is analysed [51]. Annibalini et al. [51] reported that male and female HUVECs do not express ER-α while ER-β and AR expression is similar.

On the other hand, some authors describe higher AR levels in endothelial cells from male but not from female [52,53].

The lack of significant differences in the expression of ERs genes and protein in female and male cells does not necessarily mean that the signal transduction is the same in XX and XY cells. Interestingly, NOS3 mRNA and its protein levels, which are regulated by oestrogen through both non-genomic and genomic mechanisms [15], are higher in FHUVECs than in MHUVECs. The activity of NOS3 is also increased by its phosphorylation by Akt [16]. Here, the expression of Akt protein does not present a significant sexual dimorphism in HUVECs, contrasting the previous results in other cells and tissue types [54,55].

In our opinion, the higher level of autophagy observed in MHUVECs in comparison to FHUVECs is an important result. This result has been confirmed by ultrastructural analysis showing a build-up of autophagic vacuoles at different stages in MHUVECs. These results agree with some findings obtained in other cell types and tissues, such as cardiac cells, starved rat neurons and starved embryonic rat fibroblasts and rat cardiac ventricles, where a higher autophagic response was observed in male cells or organs [24,25,56]. The male tendency to undergo autophagy could depend on increased oxidative behaviour in MHUVECs compared with FHUVECs, as suggested by the improved H_2_O_2_ production observed in male cells. The relationship between oxidative stress and constitutive autophagy is well known but quite complex [57]. Low oxidative stress may increase autophagy [57], and it has recently been observed that an increase in protein oxidation forces autophagy in cardiac ventricles of male rats in comparison with female ones [25].

Sexual divergence of autophagic processes is also suggested by the negative correlation observed between the expression of beclin-1 in MHUVECs and the weight of male newborns. According to the literature [58], the weight of male babies is positively linked to maternal weight, suggesting that the weight of the mother may exert a different influence on male and female neonates. The data also indicate that sex differences begin prenatally. Considering the hypothesis of a developmental origin of adult diseases [59], the sexual differences observed in HUVECs could be relevant in explaining the diseases of adulthood.

One of the most thoroughly studied predictors in the developmental hypothesis of diseases is birth weight: low body weight at birth is a risk factor for diabetes mellitus, while it has been reported that a high birth body weight predisposes a newborn to cardiovascular diseases [59,60]. The fact that only the weight of a male newborn is positively linked to maternal weight describes a complex scenario that suggests the relevance of sex in the hypothesis of a developmental origin of adult diseases. Considering the role of autophagy in homeostatic cell processes and in the development of diseases [61], the sex differences observed in the autophagic process in HUVECs could be one of the bases for sex differences observed in the incidence of adult cardiovascular diseases.

## Conclusions

In conclusion, these findings indicate that sex differences are present in prenatal life and are parameter-specific. This result suggests that HUVECs of both sexes should be used as an *in vitro* model to increase the quality and translational value of research, in view of the fact that endothelial dysfunction plays a crucial role in the pathogenesis of cardiovascular diseases, diabetes mellitus, neurodegeneration and immune disease in men and in women.

## Abbreviations

AR: Androgen receptor
Akt: Protein kinase B
BSA: Bovine serum albumin
ECs: Endothelial cells
ERs: Oestrogen receptors
FBS: Foetal bovine serum
FHUVECs: Female human umbilical vein endothelial cells
HRP: Horseradish peroxidase
HUVECs: Human umbilical vein endothelial cells
LC3: Microtubule-associated protein 1 light chain 3
MHUVECs: Male human umbilical vein endothelial cells
mTOR: Mammalian target of rapamycin
MTS: 3-(4,5-Dimethylthiazol-2-yl)-5-(3-carboxymethoxyphenyl)-2-(4-sulfophenyl)-2*H*-tetrazolium
NBCS: Newborn calf serum
NO: Nitric oxide
NOS3: Nitric oxide synthase 3
PBS: Phosphate-buffered saline
RT: Room temperature
SER: Smooth endoplasmic reticulum
